# Domain retention in transcription factor fusion genes and its biological and clinical implications: a pan-cancer study

**DOI:** 10.18632/oncotarget.22653

**Published:** 2017-11-24

**Authors:** Pora Kim, Leomar Y. Ballester, Zhongming Zhao

**Affiliations:** ^1^ Center for Precision Health, School of Biomedical Informatics, The University of Texas Health Science Center at Houston, Houston, TX 77030, USA; ^2^ Department of Pathology and Laboratory Medicine, The University of Texas Health Science Center at Houston, Houston, TX 77030, USA; ^3^ Human Genetics Center, School of Public Health, The University of Texas Health Science Center at Houston, Houston, TX 77030, USA

**Keywords:** transcription factor fusion gene, functional domain retention, differential expression, gene fusion network, PML-RARA

## Abstract

Genomic rearrangements involving transcription factors (TFs) can form fusion proteins resulting in either enhanced, weakened, or even loss of TF activity. Functional domain (FD) retention is a critical factor in the activity of transcription factor fusion genes (TFFGs). A systematic investigation of FD retention in TFFGs and their outcome (e.g. expression changes) in a pan-cancer study has not yet been completed. Here, we examined the FD retention status in 386 TFFGs across 13 major cancer types and identified 83 TFFGs involving 67 TFs that retained FDs. To measure the potential biological relevance of TFs in TFFGs, we introduced a Major Active Isofusion Index (MAII) and built a prioritized TFFG network using MAII scores and the observed frequency of fusion positive samples. Interestingly, the four TFFGs (*PML-RARA, RUNX1-RUNX1T1, TMPRSS2-ERG*, and *SFPQ-TFE3*) with the highest MAII scores showed 50 differentially expressed target genes (DETGs) in fusion-positive versus fusion-negative cancer samples. DETG analysis revealed that they were involved in tumorigenesis-related processes in each cancer type. *PLAU*, which encodes plasminogen activator urokinase and serves as a biomarker for tumor invasion, was found to be consistently activated in the samples with the highest MAII scores. Among the 50 DETGs, 21 were drug targetable genes. Fourteen of these 21 DETGs were expressed in acute myeloid leukemia (AML) samples. Accordingly, we constructed an AML-specific TFFG network, which included 38 DETGs in *RUNX1-RUNX1T1* or *PML-RARA* positive samples. In summary, this study revealed several TFFGs and their potential target genes, and provided insights into the clinical implications of TFFGs.

## INTRODUCTION

Chromosomal aberrations leading to gene fusions occur frequently in cancer cells. Gene fusions play critical roles in tumorigenesis, can aid in cancer diagnosis, and serve as therapeutic targets. The recurrence of a fusion gene and retention of important functional domains (FDs) are important factors in assessing whether it plays an oncogenic role and has clinical relevance. Driver fusion genes typically retain functional domains (e.g., kinase domains or DNA-binding domains) [1, 2]. In our previous study [3], we performed a comprehensive analysis of kinase fusion genes that retain kinase domains and discovered features commonly present in recurrent kinase fusion genes. In this study, we performed a systematic annotation of transcription factor fusion genes (TFFGs), aiming to identify driver transcription factors (TFs) and fusion genes (FGs) across 13 major cancer types. TFFGs may enhance the activity or result in loss of function of a TF and its target genes. TFFGs are also known for their dominant-negative effects, supported by the observation of a higher frequency of DNA binding domains than transcriptional activation domains [4].

One classical example of a TFFG is the fusion between the promyelocytic leukemia (*PML*) gene and the transcription factor, retinoic acid receptor alpha (*RARA*), which is seen in 95% of acute promyelocytic leukemia (APL) patients. The *PML-RARA* fusion protein retains domains of the RARA protein that allows binding to retinoic acid response elements (RARE) and dimerization with the retinoid X receptor protein (RXRA) [5]. This causes reduced transcriptional activation and inhibition of myeloid differentiation leading to APL [6]. Recently, the National Comprehensive Cancer Network guidelines specified arsenic trioxide and all-trans retinoic acid (ATRA) as front-line treatments for APL [7]. Pharmaceutical companies have developed many kinase inhibitors targeting kinase fusion genes; however, few drugs target TFFGs, despite their pivotal role in enhancing or reducing the functionality of a TF and its target genes. Therefore, a comprehensive analysis of TFFGs in cancer will likely provide important insights into the mechanism of tumorigenesis of TFFGs and uncover new candidate therapeutic targets.

In this study, we performed a pan-cancer annotation of 386 TFFGs including 232 TFs. Investigating FD retention led to the identification of 148 TFFGs including 109 TFs. To prioritize the potential clinical relevance of these TFs, we introduced a new scoring system, a Major Active Isofusion Index (MAII) (see Materials and Methods). We also examined binding-related FD retention and identified 83 TFFGs that retained binding related FDs, including 67 TFs. We created a prioritized TFFG network using both of the MAII scores and the observed frequency. To assess the influence of TFFGs on their target genes, we examined the differentially expressed target genes (DETGs) of the 12 TFFGs with FD retention, which occurred in at least two samples of the same cancer type. In our comparison of the expression levels of target genes in fusion-positive with fusion-negative samples in each cancer type, we found four TFFGs (*PML-RARA*, *RUNX1-RUNX1T1*, *TMPRSS2-ERG*, and *SFPQ-TFE3*) that had 50 DETGs. Interestingly, these four TFFGs had the highest MAII scores. Furthermore, these DETGs were involved in the biological processes relevant to tumorigenesis in each cancer type. Interestingly, a DETG that encodes the plasminogen activator, urokinase (*PLAU*), a known biomarker for tumor invasion, was consistently upregulated in samples positive for the four TFFGs (*PML-RARA*, *RUNX1-RUNX1T1*, *TMPRSS2-ERG*, and *SFPQ-TFE3*). Our further analysis indicated that 21 of the 50 DETGs were candidate drug targets. In addition, 14 of the 21 candidate targets occurred in samples with the *RUNX1-RUNX1T1* fusion. Finally, we constructed an AML-specific DETG network based on gene expression changes in samples with *PML-RARA* or *RUNX1-RUNX1T1* fusions.

## RESULTS

### Transcription factor fusion genes (TFFGs) retaining functional domains

**Figure 1:**
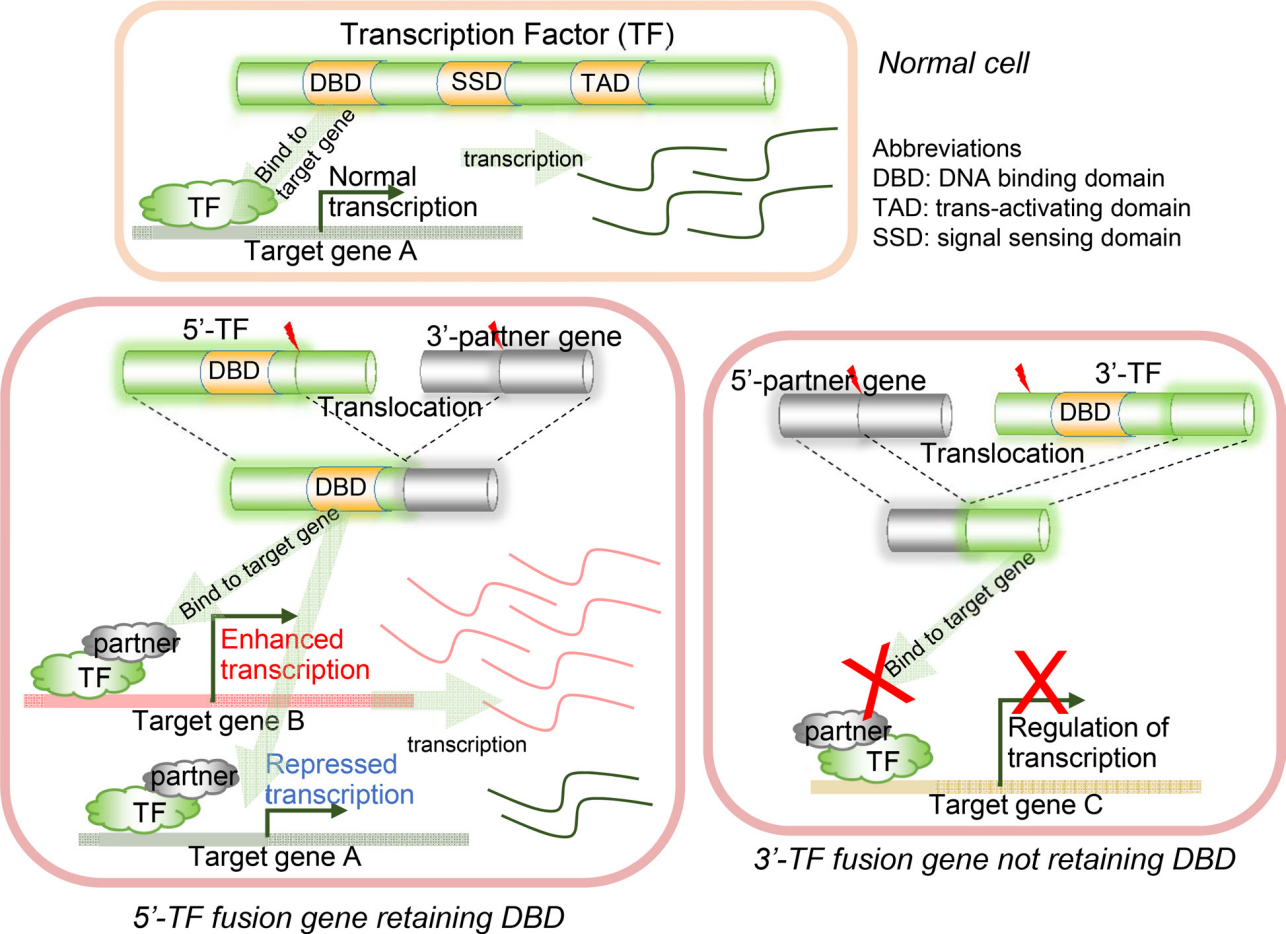
Illustration of DNA binding domain (DBD) retention in transcription factor fusion genes (TFFGs) The activities of retained domains of transcription factors (TFs) involved in fusion genes may subsequently affect the expression of their target genes.

**Figure 2:**
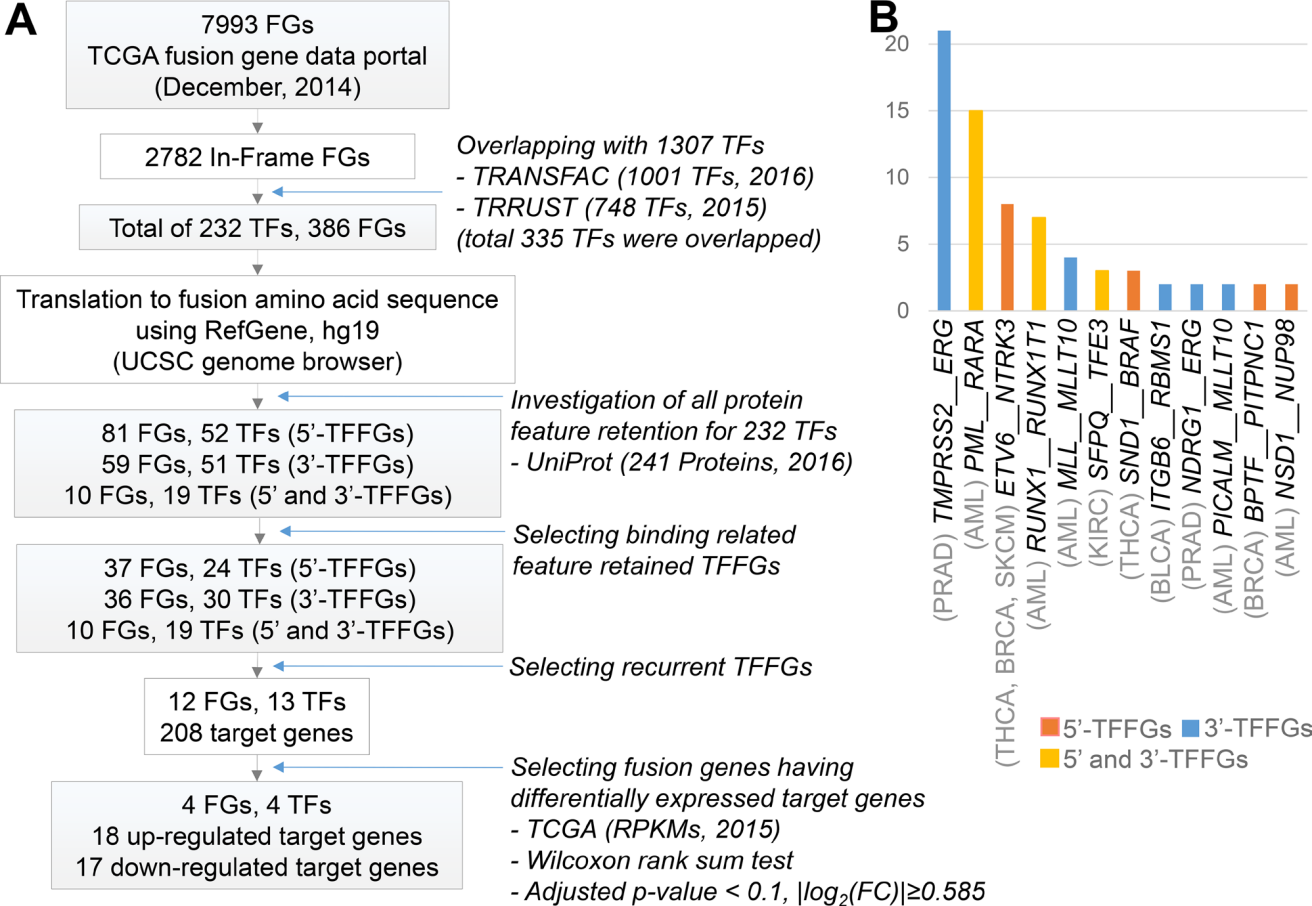
Pan-cancer analysis of TFFGs (**A**) Workflow of the functional domain retention analysis of TFFGs in pan-cancer. (**B**) Recurrent TFFGs retaining functional domains in TCGA fusion gene dataset. Y-axis represents the number of samples.

**Figure 3:**
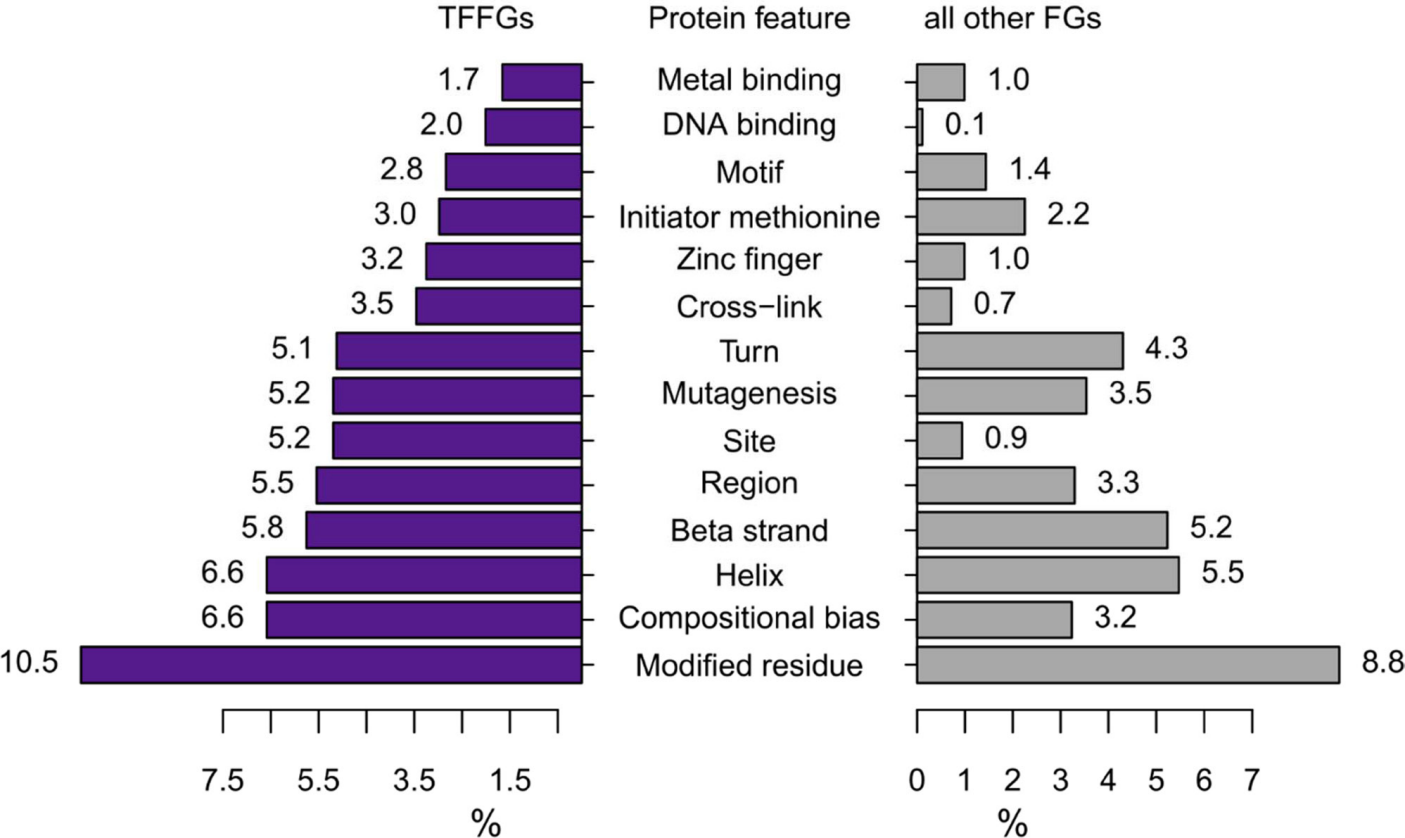
Comparison of retained protein features between TFFGs and all other FGs For each protein feature (Y-axis), the relative proportion of samples involving TFFGs and other FGs is represented. After calculating the *p*-value via a hypergeometric test, 14 protein features were selected as significantly enriched features in TFFGs, not in all other FGs (*p*-value < 0.001).

The concept of FD (i.e., fusion domain) retention in TFFGs is shown in Figure 1A. When a TFFG retains its functional domain (e.g., DNA-binding domain), the resulting fusion protein likely binds to the promoter region and the distal-regulatory region of its target genes, and regulates downstream gene expression. In contrast, if a TFFG does not retain the DNA binding domain, it would not bind to its target genes, leading to the partial or complete loss of gene expression. Figure 2A shows our pipeline for identifying driver TFs and TFFGs. From ∼8,000 fusion genes available in the TCGA Fusion Gene Data Portal [8], we selected 2,782 in-frame fusion genes. By overlapping these fusion genes with the TFs that had target gene information from the TRANSFAC [9] and TRRUST [10] databases, we obtained 386 fusion genes (FGs) involving 232 TFs. We next investigated the retention of FDs by translating the fusion transcripts into amino acid sequences and searching for the presence of 34 protein features from UniProt (see Materials and Methods). This FD retention analysis resulted in 81, 59, and 10 TFFGs that had 52, 51, and 19 TFs at the 5’-position (5’-TFFGs), the 3’-position (3’-TFFGs), or both 5’- and 3’-positions (5’-3’-TFFGs), respectively (Supplementary Table 1). To investigate which protein domains were more frequently retained in TFFGs, we compared the retention status of all UniProt's protein features in the TFFGs, with those in all other FGs (non-TFFGs). As shown in Figure 3, TFFGs significantly retained 14 out of 34 protein features at a relatively higher frequency than non-TFFGs; these domains are: ‘site’, ‘compositional bias’, ‘cross-link’, ‘zinc finger’, ‘region’, ‘DNA binding’, ‘mutagenesis’, ‘modified residue’, ‘motif’, ‘helix’, ‘turn’, ‘initiator methionine’, ‘metal binding’, and ‘beta strand’. This result is consistent with previous reports that TF fusion proteins often contain several different protein domains such as a DNA-binding domains, domains that act in homo or hetero-dimerization, and domains that interact with chromatin remodeling components such as co-repressor molecules [11]. Among these FD-retained TFFGs, we focused on the domains with TF activity such as ‘calcium binding’, ‘DNA binding’, ‘domain’, ‘metal binding’, ‘motif’, ‘nucleotide binding’, and ‘zinc finger’. After applying this filter, we identified 37, 36, and 10 TFFGs including 24, 30, and 19 TFs for 5’-TFFGs, 3’-TFFGs, and 5’- 3’-TFFGs, respectively. Only 12 TFFGs retained their FDs in at least two samples (Figure 2B). Of those, *TMPRSS2*-*ERG* was the most frequent (21 samples retained TF domains among 59 *TMPRSS2-ERG* positive samples in PRAD). Three TFFGs had a transcription factor as both (the 5’ and 3’) partners. We annotated these as 5’-3’-TFFGs. They are *PML*-*RARA* (15 samples in AML), *RUNX1*-*RUNX1T1* (seven samples in AML), and *SFPQ-TFE3* (three samples in KIRC).

### Identification of “effective” TFs in FGs by Major Active Isofusion Index (MAII) score and network analysis

**Figure 4:**
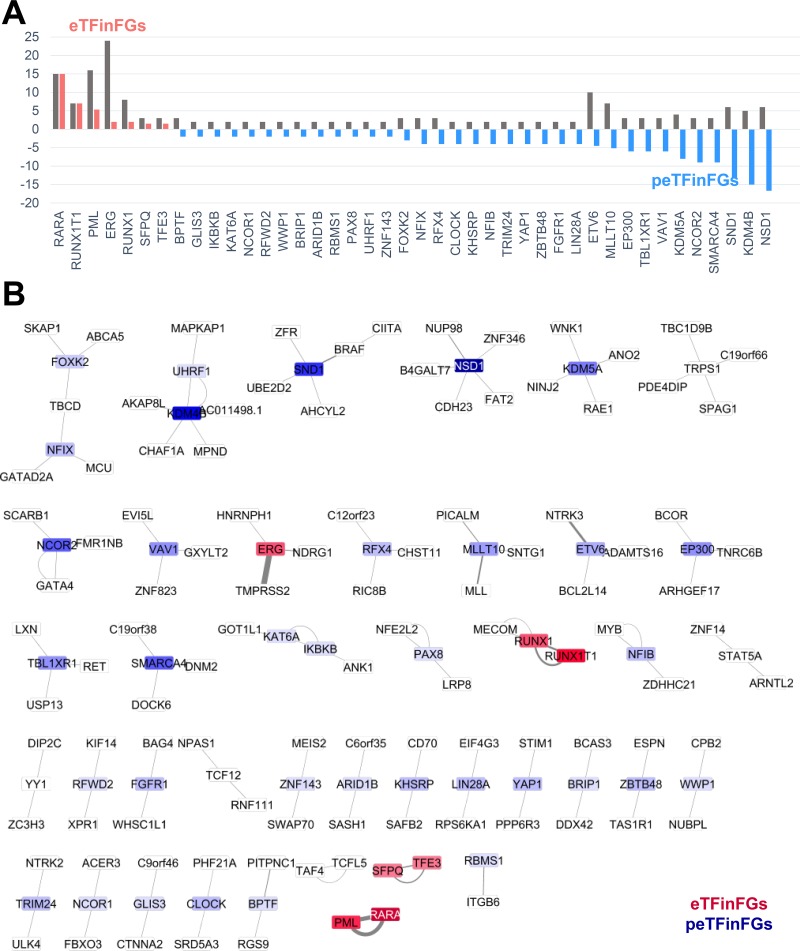
TFFG network providing clinical relevance (**A**) TFs ranked by tMAII score. “eTFinFGs” refers to effective transcription factor gene fusions (TFFGs) based on high tMAII score. “peTFinFGs” refers to potentially effective TFFGs based on low tMAII score, that is, those have higher possible combination of gene fusion than the observed frequency. (**B**) TFFG network showing TFFG pairs retaining binding domain features. In this network, we show only TFFGs including the TFs that formed fusion genes with multiple partners. Nodes in red refer to TFs with a high tMAII score and nodes in blue refer to the TFs with a low tMAII score.

We hypothesized those TFs that are involved in fusion genes in multiple cancer types, have breakpoints in multiple locations, or have multiple partner genes, would have a critical role in tumorigenesis. Therefore, we used a method we previously developed [12] to quantify the recurrence of these fusion genes. We utilized three characteristics of TFFGs: (1) the number of partner genes of each TF, (2) the number of break points in each TF, (3) and the number of cancer types associated with each TF fusion. Using these factors, we defined a Degree-of-Frequency (DoF) score (Table 1). By applying DoF scores to TFs involved in gene fusions, we found 15 potentially effective TFs including *EP300*, *ERG*, *ETV6*, *FOXK2*, *KDM4B*, *KDM5A*, *MLLT10*, *NCOR2*, *NFIX*, *NSD1*, *RFX4*, *SMARCA4*, *SND1*, *TBL1XR1*, and *VAV1*. However, in the TFFGs, the DoF scores did not always follow the observed frequency of the number of samples with fusion genes. To resolve this issue, we introduced another measurement of TF effectiveness in gene fusions: the Major Active Isofusion Index (MAII). The MAII is calculated by dividing the number of observed samples with a particular TFFG by its DoF score (Table 1). Here, an isofusion refers to one particular gene fusion combination, with one particular partner gene and one particular break point, in one particular cancer type. This new score (MAII) can give us the average frequency of each TF for each possible isofusion. A TF with a high MAII score is considered “effective” (i.e., highly recurrent) in cancer fusion genes. To make the MAII scores (ranging from 0.11 to 15) more intuitive, we transformed MAII scores of <1 to reversed negative values (tMAII). We generated a box plot of tMAII values for the TFs involved in TFFGs that retained binding domains, except those who had a DoF of ‘1.0’ in one sample (Figure 4A). A TF with a high tMAII score (i.e., >1) means that it has a high frequency of occurrence per one isofusion. We refer to these as “effective TFs in fusion genes” (eTFinFGs). The eTFinFGs include *RARA, RUNX1T1, PML, ERG, RUNX1, SFPQ*, and *TFE3*. Alternatively, if a TF has a tMAII score of less than ‘-1.0’ and a DoF score of more than ‘8’, which is the threshold of high frequent gene fusions in our previous study, it indicates that the TF has a higher chance of generating FGs in different cancer types, with multiple partner genes, and multiple break points than observed. We named these as “possibly effective TFs in fusion genes” (peTFinFGs). We found 34 peTFinFGs including *NSD1, KDM4B, SND1, SMARCA4, NCOR2, KDM5A, VAV1, TBL1XR1, EP300, MLLT10, ETV6, LIN28A*, and 22 additional genes (Table 1). Next, we created a TFFG network based on both observed frequency and tMAII scores of 83 TFFGs that retained functional domains related TF activity, including 67 TFs, (Figure 4B). In this figure, we excluded the TFFGs with a tMAII of ‘1.0’, represented by the non-highlighted cases. Using a gradient color scale of the nodes, which represents the tMAII score of each TF, we can assess the “effective TFs” and “possibly effective TFs” in pan-cancer fusion genes at a glance.

**Table 1:** The Major Active Isofusion Index (MAII)

Gene	# cancer types	# partners	# break points	DoF score	Obs. frequency	MAII	tMAII
*RARA*	1	1	1	1	15	15	15
*RUNX1T1*	1	1	1	1	7	7	7
*PML*	1	1	3	3	16	5.33	5.33
*ERG*	2	3	2	12	24	2	2
*RUNX1*	1	2	2	4	8	2	2
*SFPQ*	1	1	2	2	3	1.5	1.5
*TFE3*	1	1	2	2	3	1.5	1.5
*TRPS1*	1	4	1	4	4	1	1
*YY1*	1	2	1	2	2	1	1
*BPTF*	1	2	3	6	3	0.5	-2
*GLIS3*	2	2	1	4	2	0.5	-2
*IKBKB*	1	2	2	4	2	0.5	-2
*KAT6A*	2	2	1	4	2	0.5	-2
*NCOR1*	1	2	2	4	2	0.5	-2
*RFWD2*	1	2	2	4	2	0.5	-2
*WWP1*	1	2	2	4	2	0.5	-2
*BRIP1*	1	2	2	4	2	0.5	-2
*ARID1B*	2	2	1	4	2	0.5	-2
*RBMS1*	2	1	2	4	2	0.5	-2
*PAX8*	1	2	2	4	2	0.5	-2
*UHRF1*	2	2	1	4	2	0.5	-2
*ZNF143*	2	2	1	4	2	0.5	-2
*FOXK2*	1	3	3	9	3	0.33	-3
*NFIX*	2	3	2	12	3	0.25	-4
*RFX4*	2	3	2	12	3	0.25	-4
*CLOCK*	2	2	2	8	2	0.25	-4
*KHSRP*	2	2	2	8	2	0.25	-4
*NFIB*	2	2	2	8	2	0.25	-4
*TRIM24*	2	2	2	8	2	0.25	-4
*YAP1*	2	2	2	8	2	0.25	-4
*ZBTB48*	2	2	2	8	2	0.25	-4
*FGFR1*	2	2	2	8	2	0.25	-4
*LIN28A*	2	2	2	8	2	0.25	-4
*ETV6*	3	3	5	45	10	0.22	-4.5
*MLLT10*	2	3	6	36	7	0.19	-5.14
*EP300*	2	3	3	18	3	0.17	-6
*TBL1XR1*	2	3	3	18	3	0.17	-6
*VAV1*	2	3	3	18	3	0.17	-6
*KDM5A*	2	4	4	32	4	0.13	-8
*NCOR2*	3	3	3	27	3	0.11	-9
*SMARCA4*	3	3	3	27	3	0.11	-9
*SND1*	4	4	5	80	6	0.08	-13.33
*KDM4B*	5	5	3	75	5	0.07	-15
*NSD1*	4	5	5	100	6	0.06	-16.67

Obs: observed.

DoF score = (# cancer types) × (# partners) × (# break points).

MAII = (# obs. frequency) / (DoF score).

tMAII: transformed MAII. tMAII = if MAII < 1, then do (MAII)^-1^ × (−1).

### Analysis of differentially expressed target gene (DETG) identified consistent up-regulation of *PLAU* in four TFFGs

**Figure 5:**
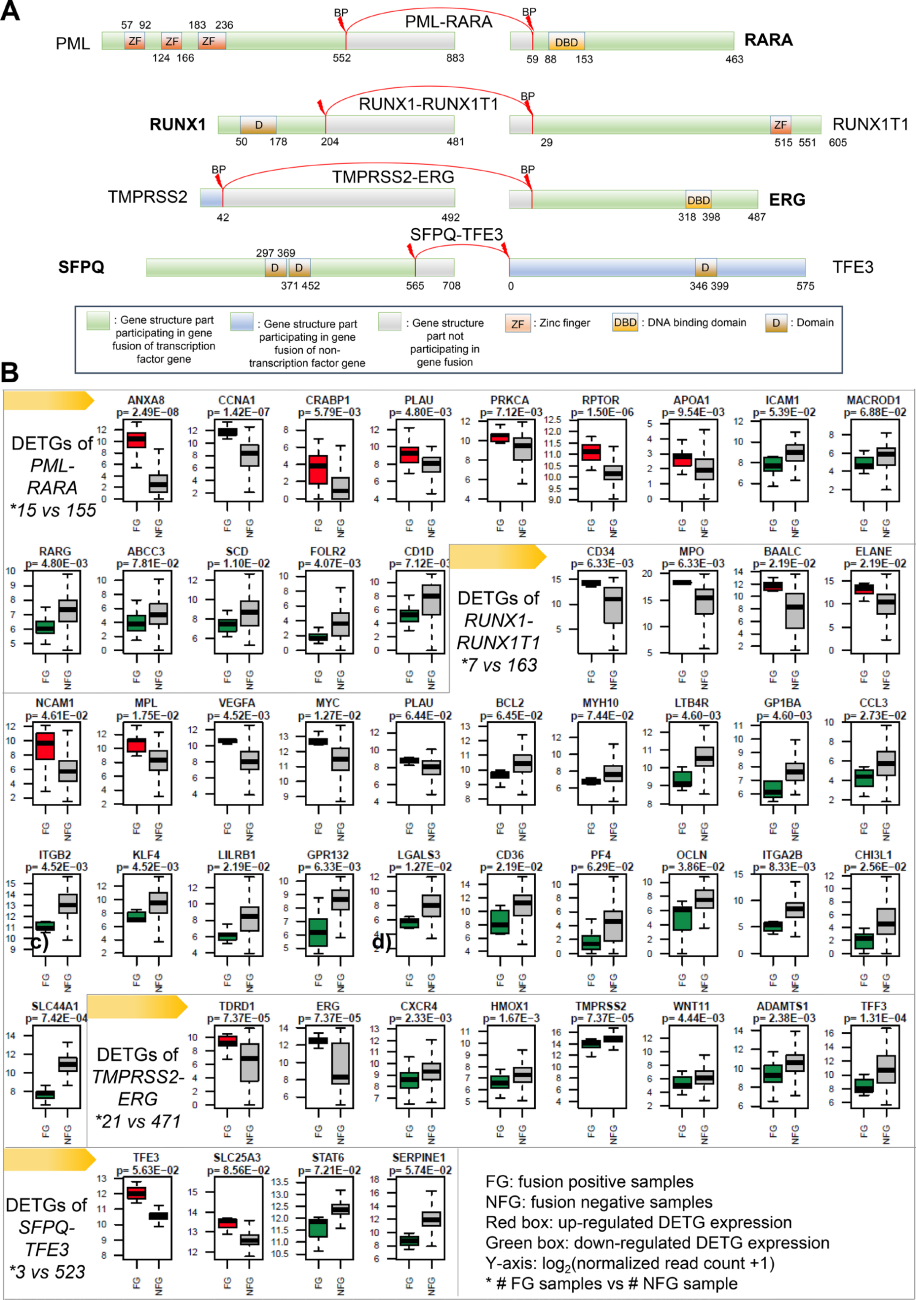
Four recurrent TFFGs and differentially expressed target genes (DETGs) in the samples with fusion genes (FGs) versus without FGs (NFG) (**A**) Fusion protein structure of the four recurrent TFFGs with highest MAII score. (**B**) DETGs of the four TFFGs in A. Y-axis: mRNA expression level measured by log_2_ (read count +1). Significantly up- and down-regulated genes in the FG samples were labeled in red and green, respectively.

Focusing on the 12 TFFGs with FD retention in at least two samples, we explored the DETGs between fusion-positive and fusion-negative samples within each cancer type (Supplementary Table 2). The aim of this analysis is to understand the oncogenic role of each TFFG in each cancer type. Analysis of DETGs (Wilcoxon rank sum test followed by multiple test correction using Benjamini-Hochberg's method [13], |log_2_(Fold change, FC)| ≥ 0.585 and adjusted *p*-value (i.e., *q*-value) < 0.1) revealed 50 DETGs from four gene fusions (*PML-RARA, RUNX1-RUNX1T1, TMPRSS2-ERG,* and *SFPQ-TFE3*). Remarkably, these four gene fusions were those with the highest tMAII scores as shown in Table 1. This supports the reliability of the tMAII scoring system in determining the biological relevance of gene fusions. A schematic representation of gene fusions and their important domains is shown in Figure 5A. Figure 5B shows the different expression levels of DETGs between fusion-positive and fusion-negative samples. In our results, a DETG is especially interesting. This DETG is the plasminogen activator, urokinase (*PLAU*, synonym: uPA), whose expression was significantly increased in AML samples with *PML-RARA* or *RUNX1-RUNX1T1* fusion genes. *PLAU* is also known to be induced by *ERG*, which is upregulated in 21 samples harboring the *TMPRSS2-ERG* fusion. Furthermore, in the *SFPQ-TFE3* positive samples, there was consistent downregulation of the serine proteinase inhibitor *SERPINE1*, an inhibitor of tissue plasminogen activator (tPA) and urokinase (uPA). *PLAU* encodes a secreted serine protease that converts plasminogen to plasmin, promoting fibrinolysis and degradation of the extracellular matrix, facilitating cancer growth and metastasis [14, 15]. As a cancer biomarker, *PLAU* plays a role in tumor invasion [16]. Recently, a positive correlation was reported between the expression level of plasminogen activator inhibitor (PAI)-1 and poor prognosis in patients with ovarian cancer [17]. These results suggest that pharmacological combination therapies using PAI-1 and urokinase inhibitors may be potentially effective in patients with *PML-RARA*, *RUNX1-RUNX1T1*, or *TMPRSS2-ERG* fusion genes.

### PML-RARA

An in-frame *PML-RARA* fusion was present in 15 of the 170 TCGA AML samples. This fusion gene retains the ‘zinc finger’ and ‘DNA binding domain’ in the 5’-partner gene (*PML*), and 3’-partner gene (*RARA*), respectively (Figure 5A). *PML* retains two different zinc finger (ZnF) domains; ZnF RING-type (PROSITE id: PS50089) and ZnF B-box-type (PROSITE id: PS50119). The ZnF B-box-type domain is considered essential but not sufficient to localize the PML protein in a punctate pattern in interphase nuclei. The DNA binding domain of *RARA* is of the nuclear hormone receptor type (PROSITE id: PS51030). *RARA* is a ligand-activated transcription factor that regulates gene expression by interacting with specific DNA sequences upstream of its target genes [18]. The up-regulated target genes of *PML*, through comparing 15 fusion-positive samples versus 155 fusion-negative samples, were *ANXA8*, *APOA1*, *CCNA1*, *CRABP1*, *PLAU*, *PRKCA*, and *RPTOR*. The overexpression of *ANXA8* has been reported as associated with AML [19]. *APOA1* is known as a biomarker for leukemia aggressiveness [20]. *CCNA1* is reported to have increased expression in AML too [21]. The down-regulated target genes were *ABCC3*, *CD1D*, *FOLR2*, *ICAM1*, *MACROD1*, *RARG*, and *SCD*. The major mechanism of tumorigenesis of the *PML-RARA* gene fusion is the disruption of the retinoic acid (RA) signaling pathway and arrest of myeloid differentiation [22]. In agreement with this mechanism, *RARG* is involved in the retinoic acid signaling and myeloid cell differentiation and *SCD* is involved in fatty acid metabolic process [23]. *ABCC3*, *FOLR2*, *ICAM1*, and *MACROD1* are involved in lymphocyte regulation, modification, and migration [23].

### RUNX1-RUNX1T1

**Figure 6:**
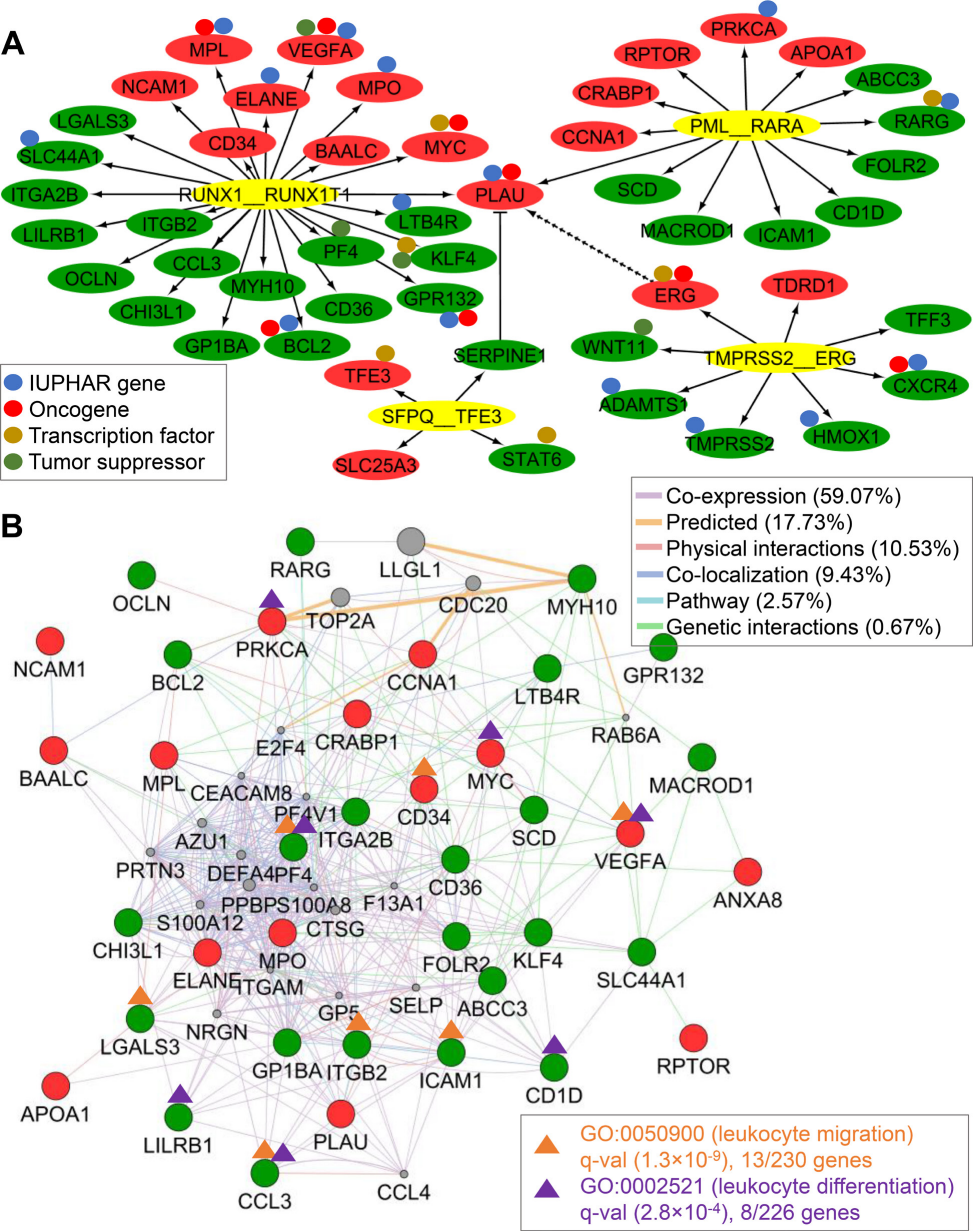
DETG network of four recurrent TFFGs and AML-specific network (**A**) DETG network of the four TFFGs. The red and green nodes represent significantly up- and down-regulated target genes, respectively. The small circles above the nodes denote drug target gene (blue), oncogene (orange), transcription factor (purple), and tumor suppressor (green) from IUPHAR [54], Catalogue of Cancer Genes [55], TRANSFAC [9] and TRRUST [10], and TSGene2.0 [56], respectively. *RUNX1-RUNX1T1* affects nine potentially targetable genes. (**B**) AML-specific TFFG network. Using 38 DETGs of the *PML-RARA* and *RUNX1-RUNX1T1* gene fusions, we created a network by using the Gene MANIA app in Cytoscape. The orange and purple lines highlight genes involved in leukocyte migration and differentiation, respectively.

Eight percent of AML and 20% of M2-type AML have this gene fusion, which is now recognized by the World Health Organization (WHO) classification system as a specific subtype of AML [24, 25]. The *RUNX1-RUNX1T1* fusion gene is known to promote self-renewal, disrupt terminal differentiation of myeloid cells, and increase DNA damage [26]. From a structural point of view, *RUNX1* retains the Runt domain (PROSITE id: PRU0039) which confers DNA binding ability [18]. *RUNX1T1* retains the zinc finger domain (MYND-type ZnF) (PROSITE id: PS50865). Proteins with MYND-type ZnF domains are known to include the transcriptional co-repressor protein BS69 within them [27]. We compared seven *RUNX1-RUNX1T1* fusion-positive samples with 163 fusion-negative samples through our DETG analysis. This analysis identified nine up-regulated genes (*BAALC*, *CD34*, *ELANE*, *MPL*, *MPO*, *MYC*, *NCAM1*, *PLAU*, and *VEGFA*). The high expression of *BAALC* and *CD34* are known as a maker for prognostic risk stratification of AML and B lymphoblastic leukemia, respectively [28, 29]. *MPL* has been reported to be involved in initiating and maintaining *RUNX1-RUNX1T1* positive AML [30]. Myeloperoxidase (*MPO*) has been associated with prognosis of AML patients [31]. Four of these up-regulated genes are oncogenes: *MPL*, *MYL*, *PLAU*, and *VEGFA* (Figure 6A). The oncogene *VEGFA*, encoding vascular endothelial growth factor A, induces proliferation and migration of vascular endothelial cells to promote angiogenesis in cancer [32]. Additionally, we found 16 down-regulated genes (*BCL2*, *CCL3*, *CD36*, *CHI3L1*, *GP1BA*, *GPR132*, *ITGA2B*, *ITGB2*, *KLF4*, *LGALS3*, *LILRB1*, *LTB4R*, *MYH10*, *OCLN*, *PF4*, and *SLC44A1*). *CCL3*, *LGALS3*, *LILRB1*, and *PF4* are involved in ‘regulation of myeloid leukocyte differentiation’ pathway. *BCL2*, *ITGB2*, and *OCLN* are the genes involving in ‘leukocyte migration’. The other genes are involved in the pathways such as ‘regulation of angiogenesis’, ‘regulation of protein kinase activity’, and ‘regulation of macromolecule metabolic process’ [23]. This result is consistent with a study of transcriptional dysregulation mediated by *RUNX1-RUNX1T1* in normal human progenitor cells and in AML [33]. While microarray data was used in this previous study, here we provided DETGs that are more accurate by using the digital expression levels from RNA-seq data.

### TMPRSS2-ERG

Chromosomal rearrangements between the androgen-regulated gene, *TMPRSS2,* and the oncogenic ETS transcription factor gene, *ERG,* occurs in approximately 30–50% of prostate cancers (PRAD) [34]. The 3’-partner gene, *ERG,* retains the ETS DNA-binding domain (PROSITE id: PS50061), which is enriched in positively-charged and aromatic residues and binds to purine-rich segments of DNA [35]. Out of the 59 *TMPRSS2-ERG* positive PRAD samples, 21 samples had an in-frame gene fusion retaining FDs. DETG analysis between 21 in-frame *TMPRSS2-ERG* positive samples versus 471 fusion-negative samples yielded only two up-regulated target genes: *ERG* and *TDRD1*. Previous transcriptional profiling studies have shown that *ERG* knockdown in *TMPRSS2-ERG* positive prostate cancer cell-lines leads to decreased expression of genes that are typically overexpressed in PRAD as compared to prostatic intraepithelial neoplasia [36]. Furthermore, *ERG* regulates the expression of target genes associated with cancer initiation and progression pathways such as DNA damage, inflammation, epigenetic control, regulation of differentiation, epithelial mesenchymal transition (EMT), cell proliferation and cell invasion [37]. Interestingly, one study demonstrated that *ERG* induced the expression of metalloproteinase and plasminogen activator pathway genes such as *MMP3*, *PLAT*, and *PLAU* [34]. The activation of the second up-regulated gene, tudor domain-containing protein1 (*TDRD1*), is known to be induced by *ERG* in prostate cancer cells harboring an *ERG* fusion [38–40].

To find DETGs that could drive cancer in the other 38 PRAD samples with *TMPRSS2-ERG* fusion not retaining functional domains, we performed DETG analysis by comparing 38 samples with out-of-frame fusion versus the 21 in-frame fusion samples (|log2(Fold change, FC)| ≥ 0.585, q-value < 0.2, and Supplementary Table 3). We found one amplified gene in the in-frame fusion samples, hydroxyprostaglandin dehydrogenase 15-(NAD) (*HPGD*), which was reported as a therapeutic target in prostate cancer due to its involvement in the arachidonic acid pathway with *PLA2G7*, *EPHX2*, and *CYP4F8* [41]. *HPGD* was highly expressed in androgen receptor (AR)–overexpressing advanced tumors, as well as in metastatic prostate cancers.

### SFPQ-TFE3

*SFPQ* retains two eukaryotic RNA recognition motif (RRM) domains (PROSITE id: PS50102). *TFE3* retains Myc-type, basic helix-loop-helix domain (PROSITE id: PDOC00038). This ‘helix-loop-helix’ (HLH) domain mediates protein dimerization. Most proteins with HLH domains have an extra basic region of approximately 15 amino acid residues and this motif sequence binds to DNA [18]. A previous study on the molecular genetics of the *TFE3* fusion gene in TCGA renal cell carcinoma samples suggested that it could contribute to carcinogenesis pathways such as *TGFβ* signaling, *MET* oncogene up-regulation, insulin signaling, Rb-dependent cell cycle, ETS oncogene regulation, FLCN/AMPK signaling, T-cell activation, and E-cadherin regulation [42]. The most widely accepted model for the oncogenic effects of the *TFE3* gene fusions is the introduction of a constitutively active promoter leading to dysregulated *TFE3* activity [16]. Accordingly, we found two up-regulated target genes (i.e., *SLC25A3* and *TFE3*) in three fusion-positive samples compared to 523 fusion-negative samples.

### AML specific DETG network

Out of the 50 DETGs identified in our study, 38 genes were from the two gene fusions (i.e., *PML-RARA* and *RUNX1-RUNX1T1*) in AML. Using these 38 DETGs as initiating genes in the GeneMANIA Cytoscape plugin [43] (see Materials and Methods, Figure 6B), we constructed an AML-specific DETG network. This network was composed of the 38 DETGs and the top 20 related genes suggested by GeneMANIA. After running GSEA for the nodes in this network (GeneMANIA, hypergeometric test followed by multiple test correction using Benjamini-Hochberg's method [13], q-value < 0.05), we found that DETGs of the AML fusion-positive samples were significantly enriched in ‘leukocyte migration’ and ‘leukocyte differentiation’ pathways, suggesting that an abnormal regulation of leukocyte function plays a role in the development of AML.

### Rare gene fusions with clinically relevant DETGs

Although we could not perform the differentially expressed gene test for TFFGs with retained FD occurring in only one sample, several examples are worth reporting. For example, Erb-b2 receptor tyrosine kinase 2 gene (*ERBB2*, synonym: *HER2*) was upregulated in one breast cancer sample containing the *ATF7-SPATS2* fusion. The expression level of *ERBB2* in the fusion-positive sample was about 25 times higher than in fusion-negative samples. Supplementary Figure 1 shows the comparison of *ERBB2* expression across 113 BRCA samples with matched normal samples, *HER2*-negative samples, and *HER2*-positive samples, according to the PAM50 annotation information for clinical subtype classification of BRCA subtype [44]. The *ATF7-SPATS2* fusion-positive sample had the highest expression level of *ERBB2* among *HER2*-positive samples and all BRCA samples.

A high level of proto-oncogene receptor tyrosine kinase, *KIT*, expression is a well-known driver of proliferation of breast cancer cells. In this study, we identified up-regulation of *KIT* in *MYB-NFIB* positive samples. *KIT* expressed 15.4 times higher in BRCA samples with the *MYB*-*NFIB* than in fusion-negative samples. The RPKM value was 13,081 in the fusion-positive sample while the average of fusion-negative samples was 849. Based on this, we hypothesized that c-Kit inhibitors might be helpful in treating BRCA patients harboring the *MYB*-*NFIB* fusion. The *MYB*-*NFIB* fusion gene resulted in loss of the 3’-end of *MYB*, including several highly conserved target sites for microRNAs that negatively regulate *MYB* expression. Deletion of these miRNA target sites may disrupt the repression of *MYB*, leading to overexpression of *MYB-NFIB* fusion transcripts and subsequent transcriptional activation of critical *MYB* target genes associated with apoptosis, cell cycle control, cell growth/angiogenesis and cell adhesion [45]. Additionally, expression of GATA binding protein 3 (GATA3), which encodes a trans-acting T-cell specific transcription factor protein, was significantly decreased in the *MYB*-*NFIB* fusion sample (RPKM was 601 in fusion sample, 13072 on average in no-fusion tumor samples). *GATA3* is one of the three genes (*TP53*, *PIK3CA* and *GATA3*) mutated in more than 10% of breast cancer samples [46].

## DISCUSSION

This study presents a novel assessment scoring system to identify TFs and FGs that may act as potential cancer driver genes, through a comprehensive analysis of functional domain retention of 386 TFFGs and their affected target genes, across 13 major cancer types. The MAII score is influenced by the frequency at which a gene fusion occurs. Therefore, the score for fusions that do not occur at a high frequency, but might be biologically relevant, could be low. A high MAII score should be better in prioritizing fusions that may be biologically significant. However, due to the lack of an independent data set with an abundant number of fusion genes across multiple cancer types, we could not extensively validate our scoring system. Furthermore, gene fusions are not as common as many of the somatic point mutations. Therefore, the small number of recurrent samples is a reflection of the nature of fusion genes in cancer. Due to this nature, we acknowledge the limitations of the DETG analysis. Another limitation of our approach is focusing on gene fusions in which at least one of the partners retained an important functional domain. Although this approach is helpful for identifying potentially active gene fusions, it cannot identify gene fusions that disrupt or eliminate the activity of a transcription factor. Thus, more investigations will be needed for TFFGs that may contribute to carcinogenesis by such mechanisms.

*ETV6-NTRK3*, a known oncogenic fusion involving a TF (*ETV6*) and a tyrosine kinase receptor (*NTRK3*), was identified as one of the 12 TFFGs with retained FDs in at least two samples, had only one DETG, growth arrest specific 2 (*GAS2*). This might be related to the fact that *ETV6* has a low MAII score, that is, the average frequency of the TF for each possible isofusion was not enough to show DETGs. In contrast, the four fusions that showed significant DETGs are those with the highest MAII scores. Furthermore, three out of four of the fusions are 5’-3’ TFFGs (*PML-RARA*, *RUNX1-RUNX1T1* and *SFPQ-TFE3*), that is, both fusion partners are transcription factors. These findings suggest that TFFGs are more likely to alter gene expression when both partners in the fusion are transcription factor genes. It is worth noting several rare TFFGs found in our study. *ERBB2* is known to be overexpressed in 18–20% of BRCA positive samples due to gene amplification [47, 48]. We identified the *ATF7-SPATS2* fusion as a potential regulator of *ERBB2* expression through *ATF7*'s action on the *ERBB2* promoter, as shown by the higher *ERBB2* expression in the fusion positive sample. Furthermore, the *KIT* tyrosine kinase gene showed up-regulated expression (15.4 times higher) in *MYB-NFIB* fusion positive BRCA. From these examples, we carefully suggest combinational therapy using kinase inhibitors to TFFG patients for better therapy.

Fusion genes are usually cancer type-specific, but TFs can be involved in multiple cancer types. Therefore, we performed a pan-cancer analysis for prioritizing TFs involved in FGs. To find the insights into the tumorigenic mechanism of TFFGs, we analyzed DETGs in each cancer type. Although the number of samples with TFFGs is small, chromosomal rearrangements involving TFs have clinical importance, due to their effects on the regulation of gene expression. This is the first study demonstrating druggable TFFGs with a systematic annotation of functional domains. A comprehensive understanding of TFFGs could help the development of new therapeutic strategies.

## MATERIALS AND METHODS

### Pan-cancer fusion gene data

The pan-cancer fusion gene dataset was obtained from the TCGA Fusion Gene Data Portal (http://54.84.12.177/PanCanFusV2, December 2014) [8]. A total of 7,993 fusion genes were curated in 13 cancer types from 4,366 primary tumor samples: bladder carcinoma (BLCA), breast carcinoma (BRCA), glioblastoma multiforme (GBM), head and neck squamous carcinoma (HNSC), kidney renal clear cell carcinoma (KIRC), acute myeloid leukemia (AML), low grade glioma (LGG), lung adenocarcinoma (LUAD), lung squamous cell carcinoma (LUSC), ovarian serous cystadenocarcinoma (OV), prostate adenocarcinoma (PRAD), skin cutaneous melanoma (SKCM), and thyroid cancer (THCA). For these fusion genes, the following information was collected: TCGA sample ID, fusion gene name and its two partner genes, fusion protein frame information, and exon junction break point information at the genomic level. We followed the definition of fusion gene direction for the 5’- and 3’-partner genes to this dataset.

### Transcription factors and their target genes

TF-target pairs were downloaded from two databases, TRANSFAC (April, 2016) [9] and TRRUST (June 2015) [10]. From the downloaded data file of TRANSFAC, we obtained 1,001 human TFs with target gene information. TRRUST is a manually curated database of human transcriptional regulatory networks. From TRRUST, we obtained 748 human TFs with their target information. Combined, we had 1,307 human TFs with target gene information.

### Annotation of protein domain retention

From ∼8,000 fusion genes, we selected in-frame fusion genes using the annotations from the TCGA Fusion Gene Data Portal. Specifically, we selected fusion genes whose reading frames were not disrupted by the breakpoints; this resulted in 2,782 in-frame fusion genes. Next, we identified the TFs and their partner pairs using the 1,307 human TFs and their target gene dataset as described above. This process led to 232 TFs that involved 386 fusion events. To survey the TF domain retention, we downloaded the protein domain annotation information for the 232 TFs from the UniProt database, using the UniProtKB search module [49]. Because the protein domain information was based on amino acid sequence, we converted the genomic break point information into the amino acid sequence by considering all UniProt protein accessions, transcript isoforms, and multiple break points for each TF. To map protein domain loci onto the human genome, we used the RefSeq gene model of human reference genome (hg19) from the UCSC Genome Browser [50, 51]. For the fusion genes whose 5’-partner genes were TFs (5’-TFFGs), we considered the protein domain being retained in the fusion if the break points were at the 3’-end of the functional domain. Similarly, for the fusion genes whose 3’-partner genes were TFs (3’-TFFGs), we considered the protein domain being retained if the break points occurred at the 5’-end of the functional domain. We also examined functional domain retention in fusion genes whose 5’- and 3’- partners were both TFs (5’-3’-TFFGs). As a result, we obtained 148 TFFGs with 109 TFs. These were 81 5’-TFFGs, 59 3’-TFFGs, and 10 5’-3’-TFFGs involving 52, 51, and 19 unique TFs, respectively. All annotations that included protein domains on the amino acid sequence for each fusion gene are provided in Supplementary Table 1. To investigate the features of TF related domains, we used fusion genes retaining binding motifs such as ‘calcium binding’, ‘DNA binding’, ‘domain’, ‘metal binding’, ‘motif’, ‘nucleotide binding’, and ‘zinc finger’ for further research. As a result, we obtained 83 TFFGs involving 67 TFs.

### Construction of the TFFG network

We built a TFFG network using gene fusion partner genes for which FDs with TF activity were retained. In this network, each node represents a partner gene or TF and each edge represents a gene fusion event. A gene fused with different partners would have multiple edges. A fusion gene can also occur in different cancer types, thus, we allowed multiple edges to represent the same fusion gene in different cancer types. We used Cytoscape (version 3.2.1) [52] for visualization and analysis of the network.

### Annotation of differentially expressed target genes (DETGs) for recurrent TFFGs

Gene expression data were obtained from TCGA (November 2016). The normalized gene expression, measured in log2 transformed normalized read count plus 1 from RNASeqV2, was extracted using the R package TCGA-Assembler [53]. The Wilcoxon rank sum test in the R software package was used in the DETG analysis followed by Benjamini-Hochberg's method [13] for multiple test correction. We defined significantly DETGs if they had |log_2_(FC)| ≥ 0.585 and *q*-value ≤ 0.1. For the expression levels of rare gene fusions, we used normalized gene expression measurements of reads per kilobase per million mapped reads (RPKM) from TCGA (January 5, 2015).

### Construction of AML DETG network

There were 38 DETGs for the *PML-RARA* and *RUNX1-RUNX1T1* fusion genes. We used these DETGs as initiating gene nodes to GeneMANIA Cytoscape plugin (version 3.4.1), a fast, in-silico, gene function prediction tool [43]. We used human network data including 20,531 genes and over 14 million interactions from the GeneMANIA database (version 2014-08-12-core). The algorithm for the construction of the network in GeneMANIA included interactions such as co-expression, co-localization, genetic interactions, signaling pathways, physical interactions, predicted interactions, and shared protein domains. Among all the network results, GeneMANIA added the top 20 related genes with up to 20 attributes using automatic weighting to the AML-specific DETG network.

## SUPPLEMENTARY MATERIALS FIGURE AND TABLES






