# Supplementary material for: Domain retention in transcription factor fusion genes and its biological and clinical implications: a pan-cancer study
Source: Oncotarget. 2017 Nov 24;8(66):110103–17. doi: 10.18632/oncotarget.22653 (PMC5746368; doi:10.18632/oncotarget.22653)
Supplement: Supplementary file 1 [file oncotarget-08-110103-s001.pdf]

Domain retention in transcription factor fusion genes and its biological and clinical implications: a pan-cancer study

SUPPLEMENTARY MATERIALS

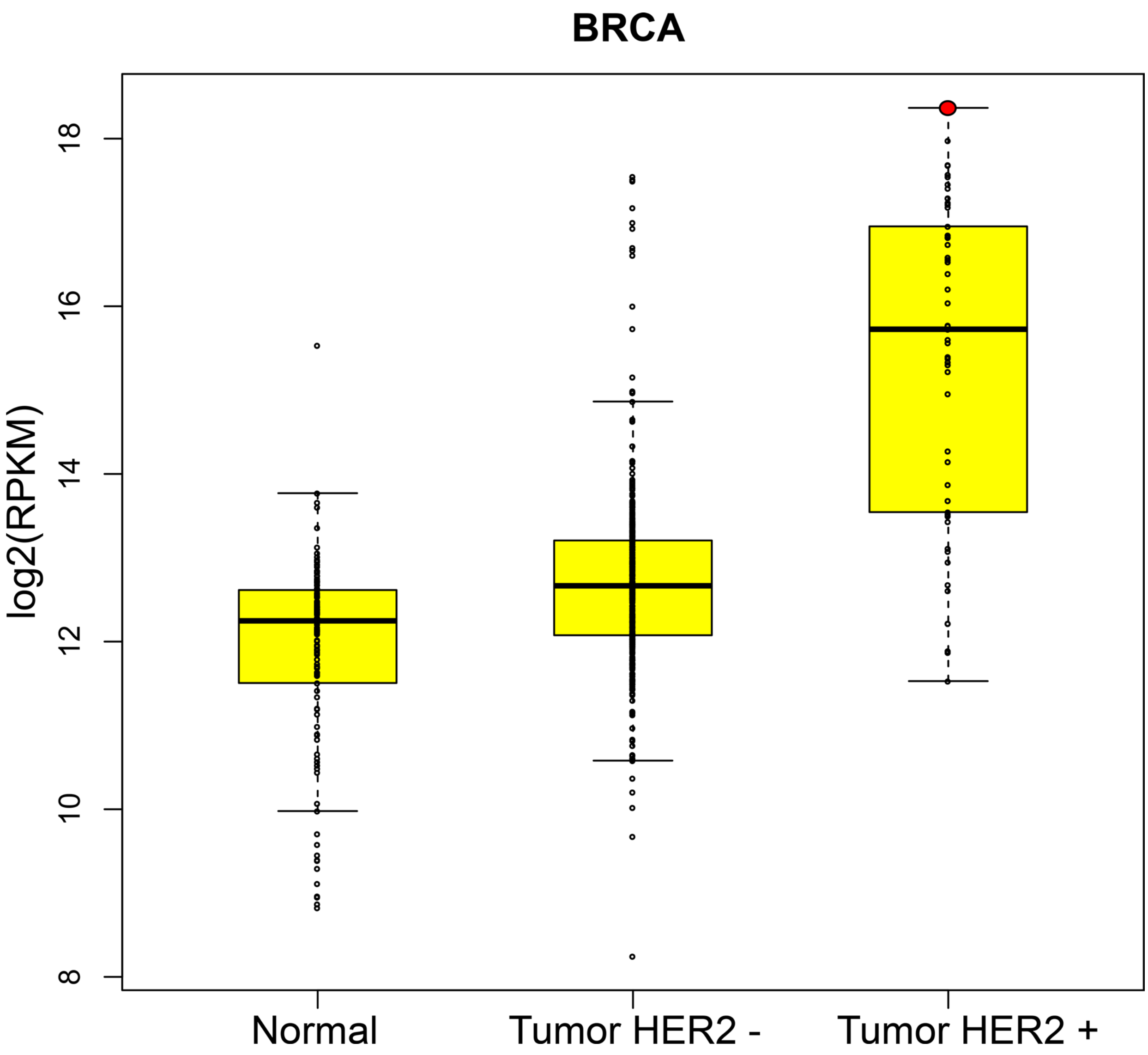

Supplementary Figure 1: HER2 expression in breast cancer (BRCA). Box plot shows HER2 expression in 113 normal samples, 413 HER2 negative samples, and 53 HER2 positive samples. The ATF7-SPATS2 fusion sample has the highest expression value of HER2 (red dot).

Supplementary Table 1: Annotation results of functional domain retention of in-frame TFFGs. See_Supplementary_Table 1

Supplementary Table 2: Differentially expressed target genes (DETGs) of 12 recurrent TFFGs. See_Supplementary_Table 2

Supplementary Table 3: DETGs of TMPRSS2-ERG (inframe vs non-inframe samples)

Fusion gene	Cancer type	Target gene	Adjs.p.val	P.val	"mean (log2(read count+1)) of fusion positive samples"	"mean (log2(read count+1)) of fusion negative samples"	log2 (fold change)	"# fusion positive samples"	"# fusion negative sample"
Inframe TMPRSS2-ERG	PRAD	HPGD	1.37E-01	5.27E-03	8.09	7.05	1.03	21	41
